# Update Advances on C-Reactive Protein in COVID-19 and Other Viral Infections

**DOI:** 10.3389/fimmu.2021.720363

**Published:** 2021-08-10

**Authors:** Ying-yi Luan, Cheng-hong Yin, Yong-ming Yao

**Affiliations:** ^1^Translational Medicine Research Center, Medical Innovation Research Division and the Fourth Medical Center of PLA General Hospital, Beijing, China; ^2^Department of Central Laboratory, Beijing Obstetrics and Gynecology Hospital, Capital Medical University, Beijing, China

**Keywords:** C-reactive protein, COVID-19, viral infection, inflammation, complication

## Abstract

Severe coronavirus disease 2019 (COVID-19) can manifest as a viral-induced hyperinflammation with multiorgan dysfunction. It has been documented that severe COVID-19 is associated with higher levels of inflammatory mediators than a mild disease, and tracking these markers may allow early identification or even prediction of disease progression. It is well known that C-reactive protein (CRP) is the acute-phase protein and the active regulator of host innate immunity, which is highly predictive of the need for mechanical ventilation and may guide escalation of treatment of COVID-19-related uncontrolled inflammation. There are numerous causes of an elevated CRP, including acute and chronic responses, and these can be infectious or non-infectious in etiology. CRP are normally lacking in viral infections, while adaptive immunity appears to be essential for COVID-19 virus clearance, and the macrophage activation syndrome may explain the high serum CRP contents and contribute to the disease progression. Nevertheless, for the assessment of host inflammatory status and identification of viral infection in other pathologies, such as bacterial sepsis, the acute-phase proteins, including CRP and procalcitonin, can provide more important information for guiding clinical diagnosis and antibiotic therapy. This review is aimed to highlight the current and most recent studies with regard to the clinical significance of CRP in severe COVID-19 and other viral associated illnesses, including update advances on the implication of CRP and its form specifically on the pathogenesis of these diseases. The progressive understanding in these areas may be translated into promising measures to prevent severe outcomes and mitigate appropriate treatment modalities in critical COVID-19 and other viral infections.

## Highlights

- CRP is normally lacking in viral infections, while adaptive immunity appears to be essential for COVID-19 virus clearance, and the macrophage activation syndrome may explain the elevated serum CRP contents.
- CRP can be used as diagnostic parameter, and also reflect the severity and prognosis of COVID-19.
- CRP is associated with CVD in COVID-19, but lacks clinical studies to support the notion that the common variation in CRP gene might have an alternative impact on the CVD.
- CRP is mainly synthesized by hepatocytes, and a small amount is produced by macrophages in inflammatory areas. How does CRP produce in COVID-19? How does CRP activate complement system and amplify the inflammatory insults?

## Introduction

The outbreak of pneumonia (coronavirus disease 2019, COVID-19) caused by severe acute respiratory syndrome coronavirus 2 (SARS-CoV-2) is a serious threat to global public health safety. The World Health Organization (WHO) has listed it as a global pandemic. As of June 4, 2021, the cumulative number of deaths has exceeded 3.7 million. There is an urgent need for effective treatment strategies. SARS-CoV-2 is highly infectious, its pathogenicity and mortality increase with age, and has strong individual differences, so it is particularly important to understand its biological characteristics. Structural analysis has elucidated the viral binding region, mutation, and host specific proteins, such as angiotensin converting enzyme 2 (ACE2) and transmembrane protease serine 2 (TMPRSS2), which are related to the entry of virus into cells and the formation of infectivity (1). Epigenetic studies have shown that DNA methylation, ACE2 gene methylation and post-translational histone modification may lead to differences in host tissue (2). NAD dependent histone deacetylase Sirtuin1 (SIRT1), could also regulate ACE2 in cell energy stress, which was upregulated in lung tissue of severe new coronavirus pneumonia patients (3). Further, cytokine release syndrome (CRS), also known as “cytokine storm”, is an important clinical feature of severe patients with COVID-19. It is characterized by the secretion of proinflammatory cytokines and chemokines, which appears to be involved in the critical and adverse prognosis of multiple organ damage and functional failure (4, 5). The identification of effective laboratory biomarkers to classify patients based on their risks is imperative in being able to guarantee prompt treatment.

C-reactive protein (CRP) is a kind of acute protein and rapidly increased in circulation in the infected conditions. In hepatocytes, CRP induction is principally regulated by interleukin (IL)-6 at transcription level, which can be enhanced by IL-1β. IL-6 and IL-1β control the expression of many acute phase protein genes by activating transcription factors including signal transducer and activator of transcription (STAT)3, C/EBP family members and Rel protein (nuclear factor [NF]-κB). The unique regulation of each acute phase gene is achieved by the specific interaction of these and other transcription factors in its promoter induced by cytokines. In addition to C/EBP binding sites, there are STAT3 and Rel binding sites in the proximal promoter region of CRP gene. The interaction between these factors makes C/EBP family members bind to DNA stably, leading to gene maximization. Extrahepatic synthesis of CRP has been documented in neurons, atherosclerotic plaques, monocytes, and lymphocytes. The latter synthesis may have physiological significance in local area but has little effect on the increase of plasma concentration. Nitric oxide (NO) can induce the decrease of CRP production through cytokine formation. As a pattern recognition molecule, CRP is usually combined with specific molecular configurations (pathogen related molecular patterns) on the surface of pathogens (6). It has certain functions similar to immunoglobulin, such as promoting agglutination, binding of complement, swelling of bacterial capsule, phagocytosis, and complex of polycation as well as polyanion (7, 8).

Of note, recent reports indicated that COVID-19 patients presented elevated CRP contents, and high levels of CRP were closely associated with more severe forms of COVID-19, where age was considered the main risk factor for poor outcome (9, 10). Likely, COVID-19 had relationship with cardiovascular disease (CVD), type 2 diabetes mellitus (T2DM), stoke, and septic complications. These diseases were also closely related to CRP levels in COVID-19. In this review, we emphasize the current state of knowledge regarding known CRP for COVID-19 and other viral infections, and potential predictive significance of CRP for organ dysfunction in patients with severe complications and poor outcomes.

## CRP and COVID-19

### CRP and Inflammation in COVID-19

Excessive inflammation is considered to be the main cause of critical illness and death in patients with COVID-19. CRP is a sensitive index to evaluate the tissue injury. Serum CRP levels are obviously increased when there are acute inflammation, major insults, and coronary heart diseases. After inflammation is subsided, it might return to normal range. It is stable *in vivo* and is not affected by trauma and hormones. CRP can recognize various pathogens and injured or necrotic cell components by combining with C-polysaccharide on the bacterial cell wall. It forms various complexes with C-polysaccharide and phospholipid and can activate the complement system in classical way to remove these pathogens and necrotic cells.

**Figure 1:**
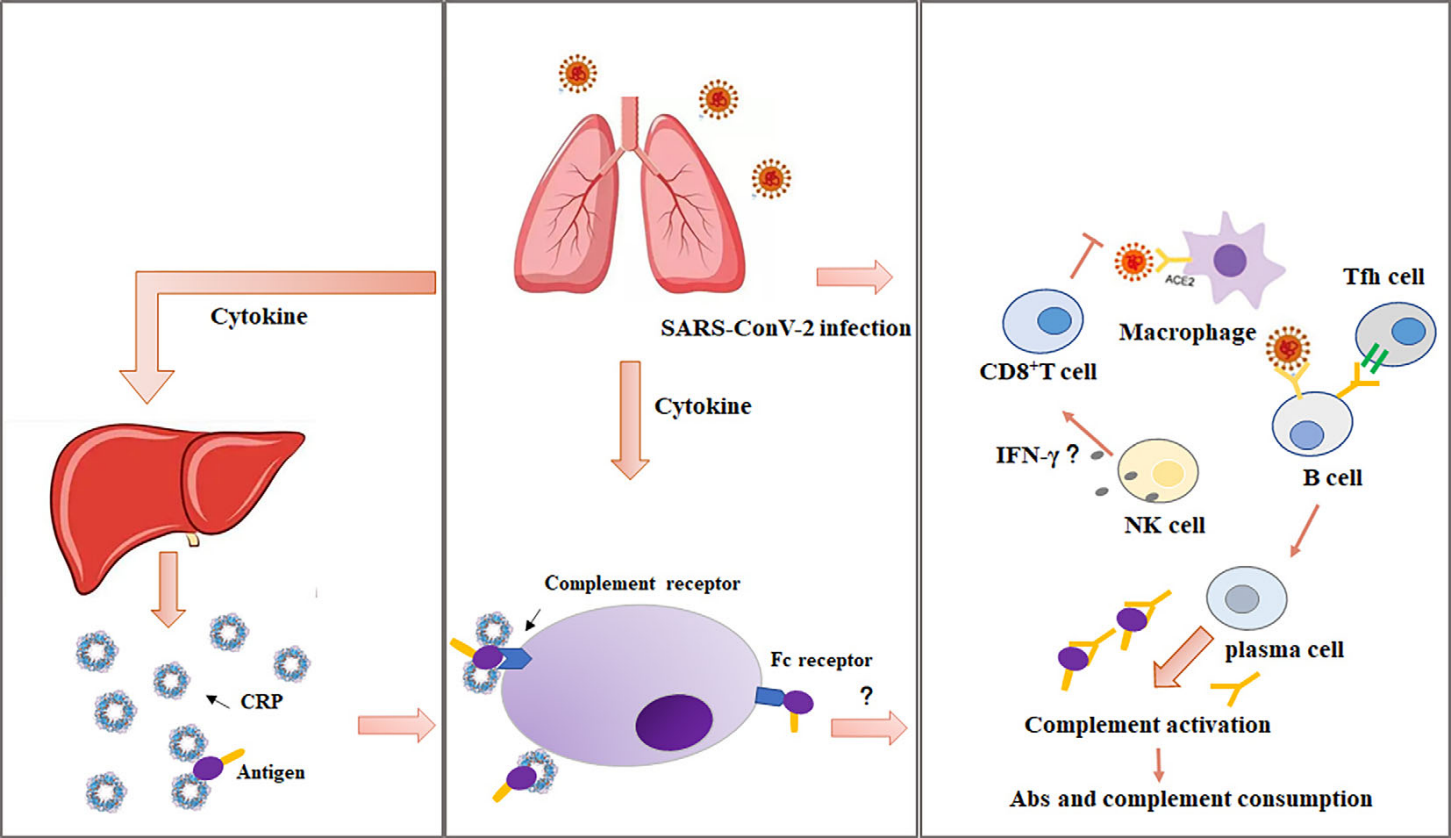
The immune regulatory mechanism of CRP in the pathogenesis of COVID-19. In the development of COVID-19, a cytokine response storm can be triggered, the cytokines such as IL-6, TNF-α, stimulate hepatocyte to produce CRP. On one hand, CRP activates complement system and amplifies the inflammatory insults; on the other hand, in severe or critical patients with COVID-19, the integrity of alveolar epithelial and endothelial barrier is severely damaged. After SARS-CoV-2 virus infection, a large number of cytokines and chemokines can be induced by alveolar macrophages and epithelial cells. During this process, adaptive immunity is difficult to start effectively due to the significant decreases in lymphocyte number together with T-cell mediated immunosuppression. Thus, uncontrolled viral infection can result in severe macrophage infiltration, further aggravating acute lung injury in the setting of COVID-19.

As we well known, CRP can enhance the phagocytosis of phagocytes through specific CRP receptor, and remove various pathogenic microorganisms. During the process of COVID-19 pneumonia, a cytokine response storm (CRS) can be triggered, which is associated with high mortality in COVID-19 (11). The cytokines such as IL-6, TNF-α, stimulate hepatocyte to produce CRP (**Figure 1**). CRP is the biomarker that most strongly correlates with COVID-19 progression, is significantly elevated during the early stage of inflammation (12, 13) and also prior to indications of critical findings with CT. Several retrospective comparison studies between survivors and non-survivors showed an increasing trend of acute-phase proteins, including CRP, procalcitonin (PCT), and IL-6, in non-survivors, and a stable or downward trend in survivors (14), CRP was verified to be independent outcome predictor and independent discriminator of disease severity (15–17), indicating that the diagnostic value of CRP for COVID-19 might be useful in clinical practice. In a multicenter retrospective study, it was reported higher level of CRP in thrombotic complication events after COVID-19 infection (18). In addition, obesity and metabolic syndrome in COVID-19 were associated with chronic systemic inflammation, including atherosclerosis and hypertension, which affected the outcomes of COVID-19 (19). A group of metabolic ill patients with obesity and COVID-19 infection showed a positive correlation with CRP (16). Likewise, the observational study of elderly Iran patients with higher body mass index by Alamdari et al. (20) demonstrated lymphopenia, hypomagnesemia, elevated CRP and/or raised creatinine on admission were at higher risk of mortality due to the COVID-19 infection. Taken together, CRP might play a vital role in the process of inflammatory response, and it can be used to assess the severity of COVID-19 and be independently associated with the risk of COVID-19.

### CRP and CVD in COVID-19

Although SARS-Cov-2 passes through the upper respiratory tract, its affinity and selective binding with ACE2 receptor cause COVID-19 to be a kind of vascular infection. Arterial or venous thrombosis is the marker of COVID-19 with CVD, which is associated with strong systemic inflammation and release of vascular injury and pro-thrombogenic cytokines. A group of CVD patients with COVID-19 revealed a consistent association between bad progression and serum CRP concentrations (21). A retrospective cohort study including 288 confirmed COVID-19 patients also demonstrated that CVD with older and higher levels of troponin I (TnI), CRP, and creatinine were more prone to develop into severe or critically severe cases (22). Moreover, 2147 patients with COVID-19 were enrolled in European, patients with cardiovascular comorbidities displayed markedly higher levels of troponin and CRP, which were markers of myocardial injury and thrombo-inflammatory activation in the uncomplicated phase of COVID-19 (23). When infected by COVID-19, patients with underlying CVD were more likely to exhibit elevation of troponin T (TnT) levels, which was a high and markedly positive linear correlation with plasma high sensitivity CRP (hs-CRP) level (24). Epidemiological studies suggested that inflammation plays an important role in various stages of CVD, including the early formation of atherosclerotic plaque, acute rupture of atherosclerotic plaque, plaque shedding leading to myocardial infarction and even death. The clinical and the basal experimental studies showed that CRP is involved in various stages of CVD through its direct influence on the pathophysiological processes such as the activation of endothelial cells and macrophages, inhibition of apoptosis of neutrophils and production of endothelial NO synthase *via* destabilizing endothelial NO synthase (eNOS), stimulation of the complement cascade (25–27). Further, CRP genetic variants is associated with the elevated mortality risk of heart diseases (28, 29). Although several studies have noticed the correlation between CRP and COVID-19 severity in patients with CVD, its potential significance in various CVD types is lacking and needs to be further investigated.

### CRP and Stroke in COVID-19

Although COVID-19, the most relevant symptom caused by SARS-CoV-2 is severe pneumonia and respiratory problems, many studies also identified other potential consequences, and some patients with severe COVID-19 are at greater risk of stroke (30). COVID-19 can result in neurological dysfunction through different ways, the brain might be damaged by hypoxia or coagulation disorders, thereby contributing to ischemic or hemorrhagic stroke. Meanwhile, SARS-CoV-2 directly infects the brain and meninges. In addition, the host immune response to infection can lead to inflammation, which can damage the brain and nerves. Several prospective studies reported that patients with COVID-19 associated ischemic stroke had severe functional outcome and high mortality (31, 32). Elevated CRP is clearly recognized in the major acute-phase response following ischemic or hemorrhagic stroke, and it is related to the development of vascular complications (33). From clinical observations, it was noted elevated concentration of D-D dimer, fibrinogen, and CRP in COVID-19 patients with acute ischemic stroke, suggesting systemic hyperinflammatory and hypercoagulable state (34). In a retrospective, observational cohort study, CRP was found to be correlated between stroke onset and the peak of acute phase reactants in COVID-19 (35). Furthermore, CRP was the mortality predictor, and its expression might be correlated with the formation of ischemia in COVID-19 associated strokes (36).

### CRP and T2DM in COVID-19

Emerging evidence from epidemiological observations, as well as reports from Centers for Disease Control (CDC) and other national health centers and hospitals, show that among patients with COVID-19, the risk of death of diabetes is 50% higher than those without diabetes, especially in elderly patients with T2DM. There is a bidirectional relationship between COVID-19 and diabetes (37). On the one hand, diabetes is associated with an increased risk of severe COVID-19. On the other hand, COVID-19 may induce new onset diabetes in normal people. During T2MD, inflammation can promote metabolic abnormalities, but metabolic factors can also regulate immune cell functions (38). The impaired immune system together with metabolic imbalance also increases the susceptibility of patients to several pathogenic agents such as the SARS-CoV-2 (38). Cross-sectional and prospective studies verified a relationship between high levels of CRP and elevated risk for T2DM (39). A comparative study revealed that symptomatic COVID19 positive T2DM patients had significantly higher CRP and absolute neutrophilic counts, lower counts of lymphocytes and eosinophils (40). Of note, many cases of T2DM with COVID-19 showed markedly increased CRP levels (41–43) (**Table 1**). Based on the multitude of clinical observations, CRP appears to be positively correlated with hemoglobin A1c (HbA1c), which is the indicator of overall glycemic control in diabetics and mortality correlation with COVID-19 (44). Further, during diabetes, many previous studies have demonstrated that CRP can bind its receptor (CD32b) to induce the inflammation process by activating Smad3-mTOR pathway, ERK/p38 MAPK or NF-κB signaling (68, 69). Collectively, serum CRP might aid in assessment the severity and improvement of T2DM in COVID-19.

**Table 1:** CRP in COVID-19 and other viral infections.

Etiology	CRP	References
**COVID-19**		
Inflammation	Positive correlation and risk factor	(12–20)
CVD	Independent predictor and severity correlation	(21–24)
Stroke	Vascular complication correlation and mortality predictor	(33–36)
T2DM	Insulin correlation and risk factor	(39–44)
Sepsis	Diagnostic value and prognostic value	(45–48)
**Other viral infections**		
SARS	Progression correlation and mortality predictor	(49–54)
MERS	Positive correlation and severity correlation	(55, 56)
H7N9/H1N1	Independent predictor and severity correlation	(57–65)
EBOV	Positive correlation and severity correlation	(66, 67)

CRP, C-reactive protein; CVD, cardiovascular disease; T2DM, type 2 diabetes mellitus; SARS, severe acute respiratory syndrome; MERS, Middle East respiratory syndrome; EBOV, Ebola virus.

### CRP and COVID-19 Induced Sepsis

From the perspective of the clinical manifestations, the essence of COVID-19 should be viewed as a sepsis induced by viral infection. COVID-19 is closely related to sepsis, which indicates that most deaths in ICU may be the direct consequence of septic complications caused by SARS-CoV-2 infection (70). The viral sepsis induced by SARS-CoV-2 has the typical pathophysiological characteristics of sepsis, that is, the early cytokine storm and the subsequent immunosuppressive stage. There are similarities and differences between severe COVID-19 and sepsis. In mild patients with SARS-CoV-2 infection, pulmonary macrophages activate the inflammatory response and phagocytize the virus, innate and adaptive immune responses can effectively inhibit the replication of the virus. However, in severe or critical patients with COVID-19, the integrity of alveolar epithelial and endothelial barrier is severely damaged. SARS-CoV-2 virus not only attacks alveolar epithelial cells, but also attacks pulmonary capillary cells, resulting in a large number of serous components leaking into the alveolar cavity. Upon SARS-CoV-2 virus challenge, alveolar macrophages and epithelial cells release a large number of cytokines and chemokines (71–73). Monocytes and neutrophils may be recruited to the site of infection and clear the exudate containing virus particles and infected cells, in turn leading to uncontrolled inflammatory response. During this process, adaptive immunity is difficult to start effectively due to the significant decrease of lymphocyte number and T-cell mediated immune dysfunction. Uncontrolled viral infection leads to severe macrophage infiltration, further aggravating acute lung injury. Simultaneously, the spread of SARS-CoV-2 can directly attack other organs, immune response contributes to systemic inflammatory storm as well as microcirculation disorders, these factors work together, and finally lead to viral sepsis. A retrospective analysis of COVID-19 deaths was performed and found that acute respiratory failure (ARF) and sepsis were correlated with disease severity and might be the main causes of death (74).

**Figure 2:**
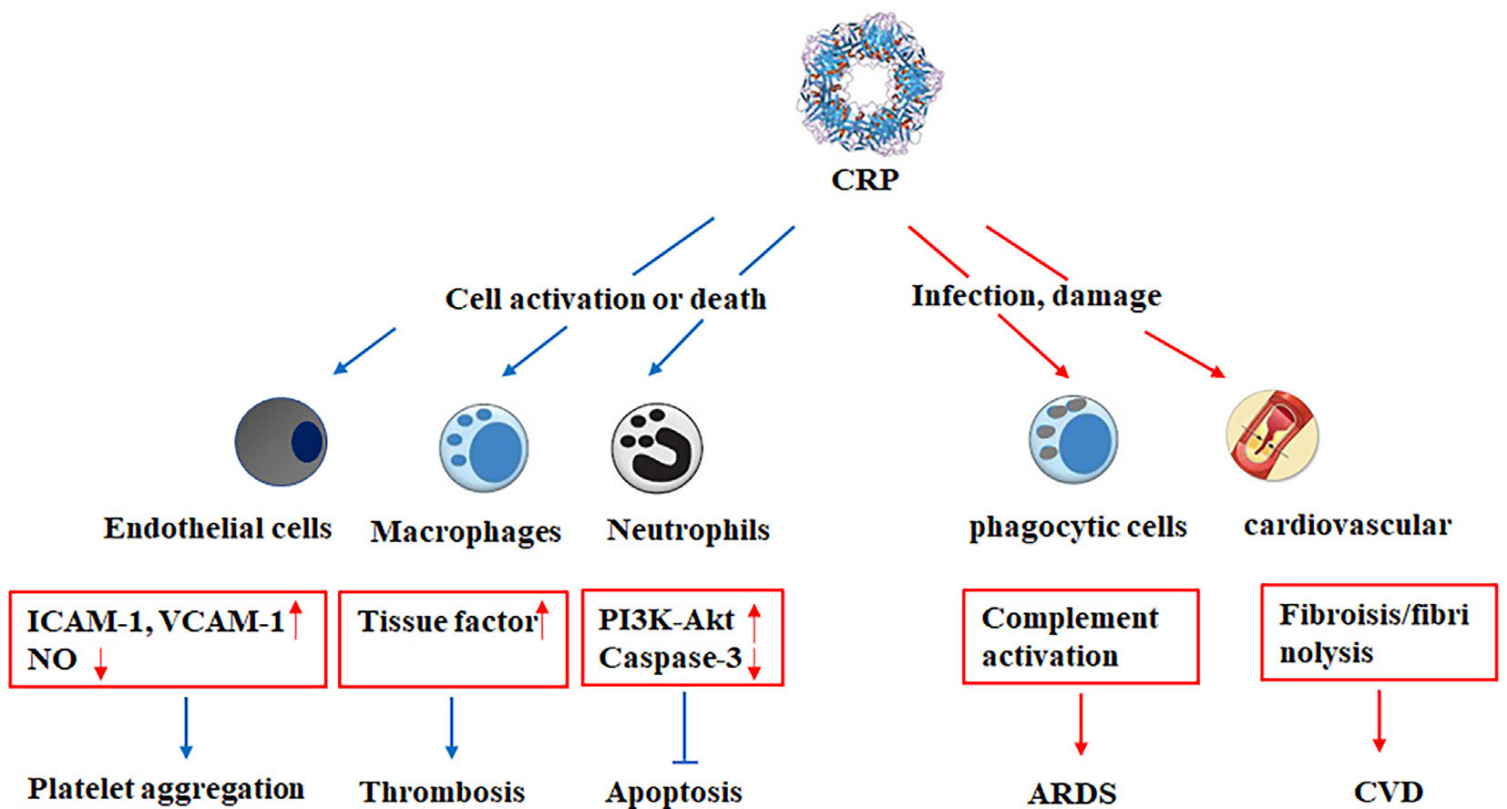
The critical role of CRP in acute or chronic inflammatory diseases. CRP may be critically involved in various pathophysiological stages of acute or chronic inflammatory diseases, including the activation of endothelial cells and macrophages, inhibition of apoptosis of neutrophils and expression of endothelial NO synthase, platelet aggregation, accumulation of lipid and thrombosis, complement activation, fibrosis, and upregulation of proinflammatory cytokine expression. ICAM-1, intercellular cell adhesion molecule-1; VCAM-1, vascular cell adhesion protein 1; ARDS, acute respiratory distress syndrome; CVD, cardiovascular disease.

Inflammatory mediators are the most active proinflammatory factors throughout the development of sepsis. At different stages of sepsis, a variety of inflammatory cytokines is noticed to be the characteristics of time-dependent release, which can reflect the condition and prognosis of septic patients. It has been documented that CRP is correlated with acute physiology and chronic health evaluation II (APCHE II) and sequential organ failure assessment (SOFA) score, which reflect the severity and prognosis of sepsis (45–47). As the part of non-specific immune mechanism, CRP can bind to Streptococcus pneumoniae capsular C-polysaccharide, bind to phosphocholine on the membrane in the presence of calcium ion, bind to chromatin, activate the complement in classical way, enhance the phagocytosis of leukocytes, and act as opsonin when stimulating the activation of lymphocytes or monocytes/macrophages (**Figure 2**). A single-centered, observational study conducted the elevated CRP, neutrophil lymphocyte ratio (NLR), and lactate dehydrogenase in non-survivors, and non-survivors were more likely to develop acute respiratory distress syndrome, CRS, and sepsis (48). Therefore, with the deep understanding of the clinical significance of CRP in the diagnosis, treatment, and prognosis of sepsis in SARS-CoV-2 infection is helpful to the early rational use of phased antibiotics.

## CRP and Other Viral Infections

### CRP and SARS

Coronaviruses have a “crown” of proteins, known as pepsomers, that protrude from the center of the virus in all directions. These proteins help the virus recognize whether it can infect the host. The disease, known as severe acute respiratory syndrome (SARS), is also associated with a highly infectious coronavirus in the early 21st century. SARS is an infectious disease with rapid onset, rapid transmission, and high mortality (75–78). Most of the infected patients are directly or indirectly contacted with patients or live in the epidemic area. The clinical manifestations are hypoxia, cyanosis, respiratory acceleration or respiratory distress syndrome, shortness of breath, and lung lesions in different degrees showed in X-ray (47, 48, 75–80). The SARS CoV-2 is the same family member as SARS, but there are differences in structure and amino acid composition between SARS and COVID-19, which is more infectious than SARS. Although only four amino acids of ACE-2, being the common receptor of SARS, are mutated, COVID-19 is 100 times more infectious than SARS. The pathological alterations of SARS and COVID-19 are very similar, while lung lesions in SARS appear to be more serious.

The lung and immune system are the main target organs of SARS and COVID-19, manifesting focal and patchy necrosis of lymph nodes and spleens, together with remarkable decreases in lymphocyte counts (81–83). COVID-19 has this kind of performance, but it is not as obvious as SARS. The prospective studies showed that increased CRP levels were associated with progressive abnormalities in patients with SARS related pneumonia and could be predictive of severity and death (49–53). Following 8 years of SARS, the association between SARS epidemic and Chinese older people revealed that community SARS exposure in the Chinese older adults was more strongly related to high CRP levels (54).

### CRP and MERS-CoV Infection

**Figure 3:**
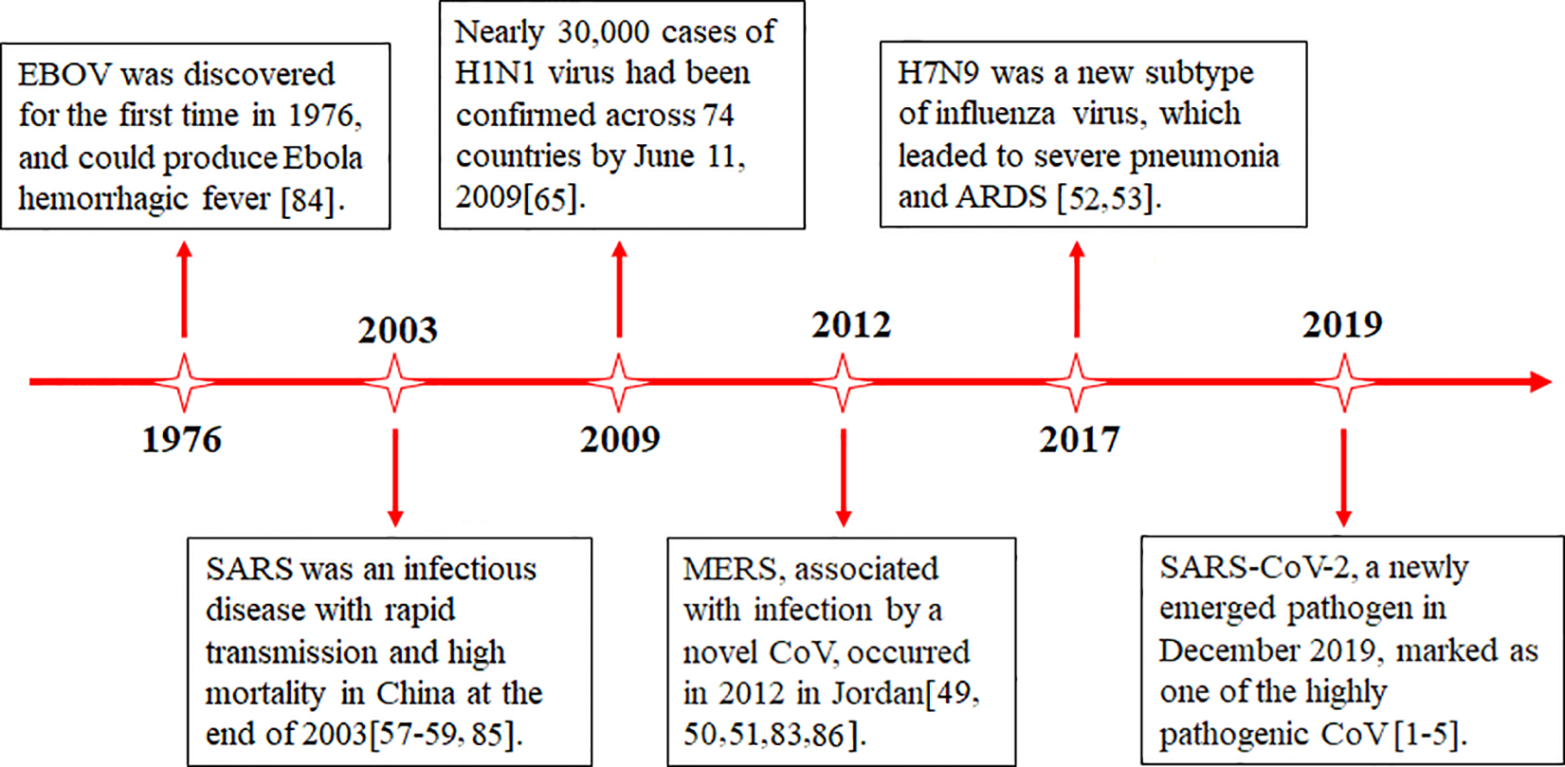
Timeline of advances in the research on various viral infections. SARS, severe acute respiratory syndrome; MERS, Middle East respiratory syndrome; ARDS, acute respiratory distress syndrome; EBOV, Ebola virus.

Since the 21st century, there have been three major outbreaks of human coronavirus, including SARS in 2003, Middle East respiratory syndrome (MERS) in 2012, and the COVID-19 pneumonia (**Figure 3**). The clinical symptoms in MERS are similar to SARS, MERS-CoV is mainly prevalent in the Middle East, but there are cases of infection and transmission in Europe, Asia, and North America. The pathological changes of MERS mainly target at both lungs, causing diffuse injury, edema, and hyaline membrane formation. It shows alveolar epithelial injury and inflammatory cell infiltration characterized by monocyte/macrophage and lymphocyte exudation. Since 2003, the research on SARS-CoV and MERS-CoV has made significant progress, including natural origins, epidemiology, pathogenic mechanism, antiviral development, and vaccine design (84, 87–90). No specific laboratory parameter related to MERS-CoV infection has been found. A retrospective observational study documented an obviously higher CRP level in MERS (55). In addition, MERS-CoV infected patients was reported to have high levels of serum creatinine, LDH, and CRP, which were correlated with severity and death (56).

### CRP and H7N9 Infection

Avian influenza virus belongs to influenza A virus of orthomyxoviridae. H7N9 avian influenza infected in human is an acute respiratory infectious disease caused by H7N9 subtype avian influenza virus. Avian influenza virus H7N9, as a new subtype of influenza virus, can cause cytokine overproduction and result in severe pneumonia and acute respiratory distress syndrome (85, 91). Compared with H1N1 virus, higher levels of CRP were induced by H7N9 virus, leading to cytokine storms. CRP levels were observed to have significantly positive relationship with several cytokines and chemokines, including macrophage chemotactic protein-1, interferon-inducible protein-10, and IL-6 (57, 58) (**Figure 2**). Strikingly, the correlation analysis of biomarkers with disease severity was performed, it was shown that plasma CRP, PCT, plasma thromboplastin antecedent (PTA) contents, and virus positive days were associated with mortality of H7N9 infection, but plasma CRP was an independent predictor of mortality in H7N9 infection (59). Likely, another study indicated that elevated CRP levels were induced and correlated with complement activation in patients infected with severe influenza A, and higher levels were induced in fatal patients (60).

### CRP and Influenza A/H1N1

H1N1 subtype of influenza A virus, known as H1N1 virus, is a kind of influenza A virus and one of the most common influenza viruses infected by human (86). Influenza A virus can be divided into several subtypes according to the antigenicity of hemagglutinin on the surface of the virus. Although there are two different types of respiratory viral pneumonia between influenza virus pneumonia and COVID-19, many clinical manifestations are similar. The biological marker such as CRP has diagnostic or prognostic value in lower respiratory tract infections and pneumonia (61, 62). Studies with regard to the relationship between CRP, oxygen exchange, and H1N1 influenza revealed that oxygen exchange and CRP could predict safe discharge from hospital (63). Retrospective observational study including 364 confirmed A/H1N1 flu patients demonstrated CRP<10 mg/dl was independently associated with A/H1N1 etiology (64), and high levels of CRP was consistently with a severe disease outcome in H1N1 influenza patients (65).

In addition, CRP might be involved in the pathogenesis of Ebola virus (EBOV) disease, which is a severe infectious disease that can cause human and other primates to produce Ebola hemorrhagic fever (92, 93). It was reported that high aspartate aminotransferase, alanine aminotransferase, CRP, and IL-6 levels were closely associated with poorer outcome of Ebola virus disease (66, 67).

## Conclusions

The large number of research papers including preprints that are published almost every day, COVID-19 spread immediately and challenged medicine, economics, and public health worldwide. Hence, early detection and isolation of suspected patients appear to be of importance in controlling such outbreak. Currently, diagnostic and monitoring methods for COVID-19 are numerous, nevertheless each of the described techniques is lack of high sensitivity and specificity. Accumulating evidence have indicated that CRP, a nonspecific marker of inflammation, is markedly associated with the severity and prognosis of excessive inflammatory responses, such as CVD, T2DM, hemorrhagic stroke, and sepsis in COVID-19 pneumonia. In this regard, CRP is not only an excellent biomarker of inflammation but also acts as a direct participant in the pathological process. Although the potential mechanisms underlying COVID-19 infection and virus-host interactions are incompletely elucidated, intensive studies on these virological profiles of COVID-19 will provide the basis for the development of preventive and therapeutic strategies against COVID-19. Furthermore, the heterogeneity of patients with COVID-19 infection suggests that multiple biomarkers should be used to evaluate the dynamic changes and treatment effect of COVID-19, and ultimately improve the clinical outcome. Finally, COVID-19 is challenging all human beings. Tackling this epidemic is a long-term job which requires efforts of every individual, and international collaborations by scientists, authorities, and the public.

## Author Contributions

All authors contributed to the article and approved the submitted version.

## Funding

This study was supported, in part, by grants from the National Natural Science Foundation of China (Nos. 81873943, 81730057), the National Key Research and Development Program of China (No. 2017YFC1103302).

## Conflict of Interest

The authors declare that the research was conducted in the absence of any commercial or financial relationships that could be construed as a potential conflict of interest.

## Publisher’s Note

All claims expressed in this article are solely those of the authors and do not necessarily represent those of their affiliated organizations, or those of the publisher, the editors and the reviewers. Any product that may be evaluated in this article, or claim that may be made by its manufacturer, is not guaranteed or endorsed by the publisher.
